# Recalling the universal health coverage vision and equity in the COVID-19 vaccine distribution plan

**DOI:** 10.11604/pamj.2021.39.197.29041

**Published:** 2021-07-13

**Authors:** Oludamilola Adebola Adejumo, Oluseyi Ademola Adejumo

**Affiliations:** 1 Department of Business Law, Faculty of Law, Obafemi Awolowo University, Ile-Ife, Nigeria,; 2 Department of Internal Medicine, Faculty of Clinical Sciences, University of Medical Sciences, Ondo, Nigeria

**Keywords:** Universal basic coverage, vaccine, COVID-19, equity

## Abstract

The COVID-19 pandemic has changed the world in so many ways since 2019 when the first case was recorded. COVID-19 pandemic has impacted negatively on economy, health, education and infrastructure globally. COVID-19 vaccine was developed with the aim of stopping the pandemic and allowing the rebuilding of our societies and economies. The vaccine was rolled out in December 2020 and the distribution plan appears to be skewed in favour of high income countries. This paper highlights the need for consideration of the principles of equity and universal health coverage in the distribution plan of the vaccine. It emphasizes the need to ensure that the interests of citizens of developing and low income countries are well protected. The paper concludes that issues of disparity in economic status of countries entering agreement with the vaccine manufacturing companies, absence of logistic support among others should not be a barrier to ensuring equitable access to vaccine for all consistent with the sustainable development goal 3.7.

## Commentary

The coronavirus disease (COVID-19) pandemic started in Wuhan, China in December 2019 and has spread across several countries. According to World Health Organization (WHO) as of 16 February, 2021, 108, 822, 960 cases of COVID-19 have been confirmed and 2, 403, 641 deaths recorded globally [1]. Although the overall case fatality is about 2.2%, the absolute number of people who had succumbed to this disease is very significant. The COVID-19 pandemic has also impacted negatively on economy, health, education and infrastructure globally. COVID-19 does not presently have a cure; however, it has been established that it is vaccine preventable. Various researchers and pharmaceutical companies have worked assiduously around the clock in the past months with different phases of clinical trials being simultaneously conducted in order to shorten the time required to develop a potent and safe COVID-19 vaccine.

Vaccines Global Access (COVAX) is a global initiative led by the World Health Organization (WHO), Gavi the Vaccine Alliance and The Coalition for Epidemic Preparedness Innovations (CEPI). This initiative brought nations together which included 192 low and middle income countries irrespective of their income level to ensure the procurement and equitable distribution of COVID-19 vaccines in order to stop the acute phase of the pandemic and allow the rebuilding of our societies and economies. They also offer guidance on the prioritization of groups for vaccination within countries while supply is limited.

**Universal health coverage:** the principle of Universal Health Coverage (UHC) was first advanced in 2015 by the international community in the Sustainable Development Goals (SDGs) agenda [2]. The SDGs was a follow up to the 2015 bound Millennium Development Goals (MDG) formulated in 2000 and set to be achieved by the United Nations. SDG 3.7 aims to achieve UHC, “including access to safe, effective, quality and affordable essential medicines and vaccines for all”. SDG 3 is an improvement to MDGs 5 and 6 which cover access only for the purpose to reducing maternal mortality and access to treatment for those with HIV, malaria and other communicable diseases. Vandervort posits that having universal access to healthcare is widely espoused as a desirable social goal in principle, but notes that some jurisdictions will have challenges relating to implementation feasibility, efficiency, conflicts and competing social goods [3]. Indeed, the practicability of achieving UHC has been tested by the recent COVID-19 Vaccination roll out plan.

**Equity of access in vaccine distribution:** events that unfolded since the first roll out of COVID-19 vaccination plan by United Kingdom in December, 2020 showed that the distribution has been skewed to Europe and Asia. In fact, as of 21^st^January 2021, only 25 doses of vaccines have been administered in Africa while about 40 million doses have been administered in higher income countries [4].

The COVAX is faced with constraints such as funding shortfall, bilateral agreement between high income countries and vaccine manufacturing companies leading to shortage of supply of vaccines to low and middle income countries, regulation and ultra cold chain requirements and concrete mass vaccination programs. These constraints may subsequently threaten global health security and economy if not well addressed. By early February, progress has been made by COVAX towards commencing the initial phase of 90 million doses aimed at immunizing 3% of the African population most in need of protection, including health workers and other vulnerable groups in the first half of 2021. The availability of these vaccines is dependent on the readiness of the countries with their vaccination program and availability of the required logistic supports to make it successful. It is also believed that more doses will be made available as production scales up. In addition, the African Union has secured 670 million vaccine doses for the continent which will be distributed in 2021 and 2022 as countries secure adequate financing [5].

One of the trending focus of the World Health Organization is to address health inequity which is a major concern in both developed and developing countries. Part of the measures to address the health inequity and ensure the closing of the equity gap in health care delivery is the principle of UHC which aims to enhance access as earlier discussed. Access in the context of healthcare is a term that is not easily defined. As far back as 1982, Daniels posited that access is a complicated notion which comprises of and is to be determined by various factors [6]. Apart from the complexities in ascertaining what constitute access, the issue of equality in the context of access to healthcare services for all is also one that has been a subject of debate over the years. Achieving health equality is an unachievable goal and thus health equity is a more achievable goal than equality.

The impossibility of approaching access from the perspectives of equality in health care, is also weakened by the fact that the principle of equal access will not necessarily guarantee equal results. This is even more so since it is unlikely at the end of the day to have an equally healthy population. Fried [7] thus opines that insisting on a right to equal access in health care is an anomaly in so far as other inequalities (in wealth, income etcetera) exists in the society. The above impracticability in achieving equality makes equity a preferred notion in the context of access to health care services. According to Braveman *et al*. health equity means social justice with respect to health and reflects the ethical and human rights concerns [8].

Equity requires concerted effort to achieve more rapid improvements among those who were worse off to start, within an overall strategy to improve everyone's health. It has been consistently argued that equity itself is hinged on fair distribution which in itself augments the theory of justice [9]. As such, access may inevitably be unequal, but not necessarily unfair. Culyer posits that an equitable distribution of health resources is that which is to the advantage of the least advantaged person [10]. This is supposed to be the case in the COVID-19 Vaccination roll-out plan. It appears impracticable to equally distribute the available Vaccines among all countries. Nevertheless, the distribution though unequal should not be seen to be unfair or inequitable and priority should be given to least advantaged countries.

Equity in health connotes striving to eliminate disparities in health between more and less-advantaged social groups occupying different positions in the social hierarchy. Although, there appears to be a somewhat general need for vaccine across countries, the issue of equity in ensuring access to the vaccine, should nevertheless be prioritized. Inequity in health service distribution will occur when individuals receive services primarily according to their place in the social structure, their enabling characteristics, or the characteristics of the health system. Factors which may jeopardize the interest of developing countries should not work to disqualify or negate equitable distribution of vaccines among developed and developing countries.

**Conclusion:** with the developments witnessed with the first roll-out, what is becoming clearer by the day is the fact that the stakeholders must revisit the roll-out plan, recall the UHC Vision and ensure equity in distribution of the vaccines not only at the international level, but also at the local distribution levels within countries. Issues of disparity in status of countries entering agreement with the vaccine manufacturing companies, absence of logistic support among others should not be a barrier to ensuring equitable access to vaccine for all. Equity of access must not be sacrificed on the altar of economic inequalities, finances or other factors which may militate against developing countries and their citizens whose internationally recognized right to health must be protected, in keeping with the spirit of UHC, and in furtherance of the SDGs.
